# Current Perspectives on the U.S. Digital Health System for Sustainable Health Care: a Narrative Review

**DOI:** 10.1055/s-0046-1816589

**Published:** 2026-11-03

**Authors:** Manijeh Berenji, Mollie R. Cummins, Ram Gouripeddi, Titus K. Schleyer, Jonathan Suh, Betty Villantay

**Affiliations:** 1 UC Irvine School of Medicine and Wen School of Public HealthIrvineCAUSA; 2 University of Utah College of NursingSalt Lake CityUTUSA; 3 Doxy.me, Inc.CharlestonSCUSA; 4 Center for Clinical and Translational Science, University of Utah Salt Lake CityUTUSA; 5 Clem McDonald Center for Biomedical Informatics, Regenstrief InstituteIndianapolisINUSA; 6 Learning Health Systems Informatics, Regenstrief Institute IndianapolisINUSA; 7 Arnold O. Beckman High SchoolIrvineCAUSA; 8 Fellow, Alma FirstLos AngelesCAUSA

**Keywords:** Climate Change, Telemedicine, Digital Health, Sustainable growth

## Abstract

**Objectives:**

This narrative review explores the role of digital health technologies in addressing climate change through four thematic pillars: mitigation, adaptation, insight, and equity. The review aims to identify current perspectives about ongoing efforts, and high-value opportunities for leveraging digital innovations to enhance healthcare sustainability and resilience, primarily focusing on the U.S. healthcare system.

**Methods:**

We conducted a narrative literature review to examine the intersections between digital health technologies and climate change strategies. We searched PubMed and Google Scholar databases using keywords including “climate change”, “digital health”, “telemedicine”, “mobile health”, and “sustainability.” We included both peer-reviewed and grey literature about climate change mitigation, adaptation, insights, and health equity.

**Results:**

Digital health technologies—such as telemedicine, mobile health apps, remote patient monitoring, electronic health records, and AI-driven analytics—offer promising pathways to decarbonize healthcare, enhance disaster preparedness, improve surveillance of climate-sensitive health outcomes, and promote health equity. However, the potential benefits are moderated by challenges, including infrastructure disparities, energy demands of digital systems, risks of greenwashing, and inconsistent real-world outcomes. Emerging initiatives emphasize developing sustainable frameworks and cross-sector collaborations to address these limitations.

**Conclusions:**

Digital health offers significant opportunities for advancing sustainable healthcare and climate resilience. However, achieving its full potential will require critical evaluation of environmental impacts, strategic investment in resilient infrastructure, and policies that ensure equitable access to technologies. Future work must integrate climate considerations into digital health innovation to build a more sustainable and inclusive healthcare system.

## 1. Introduction

Climate change poses significant threats to human health and well-being. Adverse health consequences are increasing with the number of natural disasters and extreme weather events increasing in frequency and intensity over the past few decades, seriously threatening global health [ 1 ]. It is an ongoing and accelerating crisis characterized by rising global temperatures, increasingly extreme weather events, and other environmental changes. Global near-surface temperatures exceed 1.5°C above pre-industrial levels for the first time in history [ 2 ]. Temperatures in Europe are rising twice as fast as the global average, while recent decades have seen unparalleled warming, heat waves, droughts, and water shortages. The effects of such changes have been devastating: heat-related mortality, loss of agricultural harvests, and expanded areas suitable for infectious disease transmission. With risks projected for even half-degree increments of warming, addressing climate change could not be more urgent. Additionally, climate change amplifies existing health inequities by disproportionately affecting vulnerable communities [ 3 ]. Vulnerable communities include those of low-income, marginalized racial groups, the elderly, disabled individuals, indigenous communities, and residents of developing nations. And vulnerable communities face increased risks of respiratory and cardiovascular diseases, heat-related illnesses, and vector-borne diseases [ 4 – 6 ]. Climate change also impacts mental health, leading to increased stress, anxiety, and trauma related to these events [ 4 , 7 ]. Ultimately, the impacts of climate change can disrupt or even displace entire communities.

At COP28, the United Nations Climate Change Conference held in Dubai, the first-ever Health Day was declared on December 3, 2023. One hundred and twenty-three countries subsequently signed on to the COP28 United Arab Emirates Declaration on Climate and Health, emphasizing the need for governments worldwide to prepare healthcare systems for climate-related impacts [ 8 ]. This dedicated Health Day at COP28 was significant because it brought climate and health to the forefront, spearheading the development of climate-resilient, sustainable, and equitable health systems [ 8 ]. While COP28 called for a global transition from fossil fuels, tripling renewables, and strengthening climate targets, the lack of binding commitments and cohesion to limit warming to 1.5°C is a continuing barrier to progress.

In recent years, digital health technologies have garnered attention in the climate change context. Digital health is a multifaceted approach to delivering care that encompasses the intersection of healthcare and digital technologies, aiming to improve care delivery and outcomes of healthcare services through the use of various technologies such as artificial intelligence (AI), the Internet of Things (IoT), telemedicine, wearables, electronic health records (EHRs), and mobile health applications [ 9 ]. These technologies range from formal and informal electronic health services to sophisticated tools for communication, data management, predictive analytics, remote patient monitoring, and mobile applications [ 10 ]. The implementation of these technologies extends to both healthcare professionals and consumers. When the technologies are effective, thoughtfully implemented, and well-integrated into a workflow, they support clinical care delivery, health management, and overall well-being while addressing broader healthcare access needs.

Digital health technologies (including AI-driven health applications) have demonstrated a capacity to reduce clinical carbon emissions while improving patient care and health system resilience [ 11 , 12 ]. An early systematic review found that the magnitude of greenhouse gas (GHG) emissions savings attributed to telemedicine consultations varies widely, ranging from 0.70 kg to 372 kg of carbon dioxide equivalent (CO2eq.) per consultation [ 13 ]. Remote patient monitoring can enable early detection of climate-sensitive health conditions, improving adaptation to changing disease patterns. Additionally, digital platforms can provide valuable data for climate health research and inform targeted interventions for vulnerable populations [ 14 ]. By leveraging telehealth, remote patient monitoring, and other patient-centric digital tools, healthcare systems could reduce travel-related emissions and resource consumption associated with in-person care [ 11 ].

This narrative review aims to provide the reader with current perspectives about the points of intersection between digital health and climate change adaptation, mitigation, insight, and equity. Although we reference global examples, this review primarily focuses on the United States (U.S.) healthcare system due to its significant contribution to global GHG emissions and its advanced yet uneven adoption of digital health technologies.

## 2. Methods

A narrative review was conducted to describe the intersection of digital health technologies, climate adaptation, and mitigation strategies. Narrative reviews are topical in nature and educational in intent; they do not entail systematic searches of the literature or PRISMA reporting [ 15 ]. The author team collaborated to establish the objective and scope of the review, identify key topics for discussion, and outline the manuscript. Individual authors assumed primary responsibility for identifying and reviewing literature related to mitigation, adaptation, insight, and equity. Authors searched PubMed and Google Scholar using keywords that included climate change, digital health, telemedicine, mobile health, and sustainability, as well as additional keywords as needed. We selected articles relevant to the four core themes—mitigation, adaptation, insight, and equity, and referenced additional grey literature, including World Health Organization (WHO) resorts and national studies as appropriate. Analysis involved narrative synthesis highlighting intersections between digital health applications and climate challenges.

## 3. Results and Discussion

### 3.1 Mitigation: Digital Health and Decarbonization Strategies

The healthcare sector plays a significant role in climate change, responsible for about 8.5% of GHG emissions in the U.S. and as much as 4.6% worldwide [ 16 ]. Health systems directly control facility emissions, such as on-site boilers and electric usage. They may influence, rather than control, the indirect emissions stemming from supply chains (incl. transporting pharmaceuticals, supplies, and other medical products and devices). More than 80% of overall healthcare emissions in the U.S. arise from indirect sources within these supply chains [ 17 ].

Although this sheds light on the emissions tied to the internal operations of the healthcare system, it is important to note that emissions related to patient travel are largely unmeasured. As the population ages and chronic diseases become more prevalent, the demand for healthcare services, particularly inpatient care and rehabilitation, is expected to rise significantly in the U.S. [ 18 ]. Moreover, while the global health workforce is projected to expand by 23%, growing from 43.5 million in 2018 to 53.9 million by 2030, it still falls short of the estimated need for 80 million health workers [ 19 ]. There remains an 18 million shortfall, primarily in low- and middle-income countries, which will increase the need for both patient and paramedic transportation. This shortfall underscores the healthcare industry’s critical role in contributing to air pollution.

In light of these factors, digital health tools—particularly telemedicine—are compelling as a means to enhance care delivery and reduce environmental impact. Retrospective analyses of patients participating in outpatient specialty consultations through telemedicine in the U.S. have shown a positive effect of telemedicine on emissions reductions in ambulatory visits [ 20 ] and patient-related travel [ 21 – 23 ]. In Canada, a pilot prospective observational study conducted in a remote First Nation community in northeastern Saskatchewan demonstrated that remote presence robotic technology led to a decreased need for specialized pediatric interfacility transportation. This implementation eased access to care and lowered costs for the Canadian healthcare system [ 24 ].

Although 9.78% of GHG CO2 emissions in the transportation sector declined throughout the COVID-19 pandemic in the U.S. [ 25 ] compared to 2022, there was a variation in the distribution of air pollution related to traffic between urban and rural regions across the U.S. [ 26 ]. Consistently, urban neighborhoods characterized by lower income, limited educational attainment, and higher proportions of minority residents are exposed to elevated levels of nitrogen dioxide (NO₂) across the U.S. in nearly all of the 15 largest metropolitan statistical areas. Among rural communities, those with greater income and educational attainment tend to have higher levels of NO₂ than their lower-income, less-educated counterparts. Reducing environmental inequities will require forthcoming policies with a more comprehensive approach, addressing passenger vehicle emissions and incorporating emissions from surrounding sources. A key step includes tracking patient travel-related emissions in healthcare, similar to efforts led by the National Health Service [ 27 ] to differentiate from road transportation GHG emissions in the case of the European Union, which had a decline of 9.93% in GHG emissions throughout the COVID-19 pandemic compared to 2022 [ 28 ].

Digital technologies bring numerous advantages; however, they also require significant amounts of electricity, which is still generated from fossil fuels, leading to increased carbon emissions. Electricity generation in the U.S. healthcare sector accounts for about 11% of GHG emissions [ 17 ]. Consequently, there have been concerted efforts to shift paradigms in healthcare to mitigate environmental impact. Initiatives like “Green Hospitals” [ 29 ], “green-chips” [ 30 ], and comprehensive health informatics frameworks [ 31 ] have emerged in this context.

Despite evident progress, the ongoing development and validation of effective frameworks and assessment tools to measure the environmental impacts of digital health interventions remain crucial areas needing attention [ 31 ]. Tackling these varied challenges will require collaborative policymaking, user-friendly technology design, education in digital health, and ongoing evaluation to foster a more equitable and environmentally sustainable healthcare system.

### 3.2 Adaptation: Digital Health Response

As defined by the Intergovernmental Panel on Climate Change, climate adaptation in human systems is “the process of adjustment to actual or expected climate and its effects, to moderate harm or exploit beneficial opportunities” [ 32 ]. For healthcare systems, adaptation strategies are crucial for moderating climate change’s harmful health effects and ensuring operational continuity and quality. These strategies take various forms, addressing surge capacity and continuity of health care during natural disasters and the care of climate-change-related illnesses, including infectious disease outbreaks, malnutrition, and smoke inhalation. Digital health and telehealth technologies play a crucial role in healthcare climate adaptation. These technologies are instrumental in augmenting prevention or early intervention of climate-related illness, aiding individuals, communities, and health care providers in minimizing harm. They are also essential for ensuring continuity of care and increasing capacity during surges in demand for health care services during extreme weather events and natural disasters.

#### Preventing or Limiting Exposure.

Both healthcare providers and consumers with smartphones can access a plethora of applications (apps) that provide real-time information and forecasting related to outdoor temperature and air quality. Heat and air quality alerts enable individuals to make adaptive choices. When employed by individuals and communities, these apps can be used to identify and avoid individual exposures to heat and air pollutants. Community events can be rescheduled, employers can implement telework for those eligible, and optional outdoor activities can be curtailed in the interest of public health. Individuals can relocate to safer locations when feasible.

Additionally, healthcare providers can adapt their programs and services. For example, they can issue alerts and guidance to patients using patient portals and enable telemedicine appointments when it is safest to stay home. However, literature describing such climate-adaptive actions is sparse. Some evidence indicates that individuals avert exposures in response to awareness of exposure risks. For example, a 2024 study showed reduced park and playground visitation during air quality events [ 33 ]. An earlier study in China showed that individuals reduce outdoor activities in response to increased air pollution [ 34 ]. Data from a bike-sharing service in Argentina indicates that there are decreases in outdoor cycling after heat warnings [ 35 ]. Additionally, multiple studies have related heat warnings to lower mortality.

However, other evidence indicates that awareness of exposure risk does not translate to action or improved outcomes. A 2013 systematic review suggested that people largely do not act in response to heat warnings and noted that averting behavior is likely affected by costs and other barriers to action [ 36 ]. Additionally, a 2018 study determined that heat alerts are not associated with lower mortality in most US cities [ 37 ]. The authors point to the varied evidence for heat warnings and the potential importance of individual and community action plans. Centers for Disease Control (CDC) guidance notes that most people are aware of heat alerts when issued but may not change their behavior accordingly [ 38 ]. The integration of digital health technologies is of paramount importance in this context. Digital health platforms facilitate real-time environmental monitoring and dissemination of early warnings, enabling individuals and communities to make informed decisions that minimize exposure to hazardous conditions. These technologies also support healthcare system resilience by integrating environmental and health data, thereby enhancing surveillance and adaptive responses to climate-related health threats.

#### Infectious Disease Surveillance

Through multiple mechanisms, climate change is causing an increase in mosquito-borne infectious diseases, including malaria and dengue fever [ 39 ]. Digital health approaches to surveilling, forecasting, and addressing increases in mosquito-borne disease are foundational to adaptation. The WHO considers surveillance a core malaria intervention [ 39 ] using digital health technologies heavily, as fundamentally enabling investment, resource allocation, and evaluation of public health interventions. Their widespread adoption is essential for closing gaps in healthcare access and accelerating progress toward disease control and elimination. As technology continues to advance, leveraging these innovative solutions will be vital for building resilient healthcare systems capable of responding to current and future public health challenges [ 40 , 41 ].

#### Natural disasters

Climate-related extreme weather events can destroy communities and cause large-scale morbidity and mortality. These natural disasters trigger a surge in demand for health care services, even as facilities and infrastructure for providing health care may have been destroyed. Varied digital health technologies can be used to prepare for these natural disasters and during response and recovery.

Telemedicine has been applied in disaster response since the 1980s, and multiple formal telemedicine programs for disaster planning, response, and recovery exist [ 42 – 45 ]. In 2017, NATO established the Multinational Telemedicine System (MnTS), an emergency telemedicine program designed for emergency services and medical care across four Eastern European nations. The MnTS accounted for both the communication systems and power sources needed to support telemedicine, as well as the necessary hardware and software in a single, fully integrated system for use by emergency teams. In 2017, during Hurricane Irma, Nemours CareConnect, a telemedicine program, provided pediatric telemedicine services directly to consumers without intermediation by disaster teams [ 46 ]. Currently, there is an unprecedentedly high level of preparedness to use telehealth, given widespread adoption, infrastructure, and user acceptance due to the growth in telehealth during the COVID-19 pandemic [ 47 ].This makes telehealth a potentially powerful modality for delivering care in the wake of a natural disaster.

Despite this promise, telemedicine has potential limitations in disaster scenarios [ 48 ]. Connectivity can be poor in the wake of destructive natural events, and the use of telemedicine can depend upon the deployment of dedicated telecommunication resources (typically via the military or government) or the rapid restoration of power and telecommunications. In the future, as telemedicine and telehealth applications become increasingly dependent upon high bandwidth and integration of AI powered features, their performance under limited bandwidth and low energy conditions may degrade. In the U.S., issues of reimbursement, credentialing, and licensure may restrict the use of telemedicine during natural disasters [ 49 ].

Digital health solutions are increasingly powerful in climate adaptation. They play a prominent role in current surveillance and response efforts and offer individuals and communities early warning and risk assessment. However, there is a need for more insight into the effective design of digital health applications intended to effect behavior change and how healthcare providers, systems, and communities can better leverage digital health technologies to prevent or limit harmful exposures. In planning for the use of telemedicine during natural disasters, interoperability and the sharing of health information are essential. Additionally, digital health solutions must be able to perform under conditions of limited bandwidth and power. Efforts to digitally adapt healthcare systems to climate events must be mindful of equity considerations to ensure marginalized populations, who disproportionately bear climate-related health impacts, benefit from technological advancements.

### 3.3 Insight: Understanding the Effects of Climate Change on Health

The effects of climate change on health can be broadly classified as either directly or indirectly affecting health [ 50 ]. Direct effects of climate changes can include those due to extreme weather events of heatwaves, floods, hurricanes, and wildfires [ 51 , 52 ]. Indirect effects include those that directly affect environmental, ecological, and agricultural systems, and in turn affect human health and well-being [ 51 – 53 ]. These include air, water, and soil quality, conditions with infectious etiopathologies, especially vector-borne diseases, and food and water security. These then have effects on social systems, leading to poverty and displacement.

Climate effects are unevenly distributed, spatially and temporally. Vulnerable populations, such as those in low-income regions, have lower climate resiliency and are at greater risk [ 54 ]. Sudden and extreme changes in weather, such as the Pacific Northwest heatwave, have been implicated in excess mortality and morbidity [ 55 , 56 ]. Additionally, chronic exposure to heat has been shown to accelerate epigenetic aging [ 57 , 58 ]. While there are significant variations in mortality due to floods, hurricanes, and wildfires, partly due to modern weather forecasting and safety measures, various systems that humans depend upon are increasingly affected [ 59 – 61 ]. Air quality can have immediate and long-term effects and can arise due to natural, wildfire, desertification, and anthropogenic causes [ 62 – 63 ]. Urban systems and indoor environments can be traps for the effects of climate change [ 64 ]. Emergent infectious conditions and vector-borne diseases have shown a consistent shift in patterns related to climate [ 65 – 69 ]. Increased chronic conditions, including auto-immune disease, can be attributed to climatic infections and environmental patterns [ 70 – 75 ]. Yet, greater impacts of climate change are to come [ 76 , 77 ].

Quantifying the effects of climate on health needs to consider the complex interactions of various systems at different scales. These multilayered, sequential, intermittent, and cooperative stressors have immediate and delayed effects leading to the development of acute and chronic conditions [ 78 ]. Moreover, some of these effects can differentially be measured at individual and population levels. The analysis of future effects requires predictive and simulated analysis approaches [ 79 ]. Traditional approaches analyze individual stressors and exposures on health, and with limited spatial and temporal resolution. A complete understanding of climate on health needs to consider the combinatorial record of exposures and interactions as in a ‘One Health’ paradigm [ 80 , 81 ]. Such a framework consists of data from sensors, clinical encounters (EHRs and study collections), patient-reported and generated activities, computational models, analyses of biospecimens, and geographic aggregates – broadly digital health [ 82 ].

Digital health methods and technologies are increasingly being explored as tools to assess the effects of climate change on human health [ 14 ]. Digital technologies provide solutions for sensing, monitoring, electronic data capture, modelling, decision support, and communication tasks [ 83 ]. The evolving landscape of digital health technologies can generally be classified as those that capture changes in: (1) environmental, ecological, agricultural, and human systems, and (2) responses in humans, both at individual and population levels [ 84 , 85 ]. The first category consists of a broad array of sensing technologies, including various low-cost sensors, sensor networks, citizen networks, IoT devices, environmental monitors, and satellites [ 86 , 90 ]. Human and specifically health data is available through wearables, personal health devices, EHRs, telemedicine records, mobile health apps, and electronic data capture systems [ 91 – 96 ]. In addition, various indices at aggregate geographic levels, such as the social vulnerability index, environmental justice index, and climate indices, are useful metrics for vulnerability and resilience of populations and systems to climatic effects [ 97 – 102 ]. While there is an increase in data availability from these digital technologies, there are still varied levels of availability in rural and urban areas and vulnerable areas [ 103 ].

The heterogeneity of digital health data needed for quantifying the effects of climate on health requires various informatics methods. Each domain has its semantics, and individual resources within a domain usually have their own approach to representing data. For example, sensors and wearable devices use a range of metadata and data standards, and there are ongoing efforts in harmonizing sensor metadata for exposure health research [ 104 ]. Also, each of the domains represents different root objects ranging from persons, homes to geographic regions, requiring novel event-based formats for integrating such data [ 105 ]. Such a representation can then be used to reason between conditions and changes occurring in humans and those in various systems to understand causes and effects. Integrating these data needs to account for the possibility of supporting diverse study designs requires metadata-driven Big Data technologies in software ecosystems [ 106 ]. Analysis of these data can be non-hypothesis driven and include machine learning methods for ascertaining sequences of exposures, shapes of spatial and temporal patterns of exposures, and predictions of health outcomes [ 107 – 110 ]. In addition, given the possibility of gaps in data, there is a need to quantify the quality and uncertainties associated with the data and predictions [ 111 , 112 ]. Many scenarios related to human behaviors and mobility pattern analyses are suitable only for what-if simulation using agent-based and other computational modeling approaches [ 113 – 115 ]. Different methods to understand the climate change effects include multiscale modeling and digital twins of environmental and ecological systems [ 116 , 117 ]. These advanced analytical methods derive insight from various digital health data to enable timely detection of changes, estimate risk, and predict future states for individuals, populations, and systems at high spatial and temporal resolutions for current, mitigated, and adapted scenarios [ 14 , 118 ].

The human impacts climate change, as it affects disease onset and well-being, is a complex mix of extreme events with acute effects and larger systemic events occurring over larger temporal and spatial scales [ 119 ]. Digital health technologies provide essential data for understanding these effects, but there are insufficient data, informatics, and analytical gaps, necessitating foundational work in quantifying health effects of climate [ 120 , 121 ]. Exposure health informatics methods can derive promise from the current varied digital health landscape [ 122 ] for an improved understanding of climate effect on health and accelerate innovation for mitigation, adaptation, and resiliency [ 123 ].

### 3.4 Equity: Health Inequities and Climate Change

In the U.S., areas with higher proportions of African American and Hispanic residents are typically hotter due to the urban heat island effects caused by limited tree cover. These communities also have much less access to technology, such as air conditioning, increasing their vulnerability to future heat waves. Urban heat islands disproportionately impact marginalized communities, with summer daytime surface urban heat island (SUHI) intensity analysis revealing racial and economic disparities across 175 major U.S. urban areas [ 124 ]. The average person of color (including those of African American and Hispanic American descent) faces higher SUHI exposure than non-Hispanic White residents in 97% of these regions, while poverty-line households experience heightened heat compared to those in higher socioeconomic brackets. This gap was not fully explained by income, as racial disparities persist even when controlling for poverty. Though age demographics show minimal differences, systemic inequities in heat exposure disproportionately burden communities of color across all age groups, underscoring structural drivers of climate-related health risks beyond individual socioeconomic factors. As a result, heat-sensitive illnesses, such as asthma and cardiovascular conditions, are much more prevalent in these populations [ 125 ]. African-American women face much higher risks of preterm birth (PTB) during heat waves compared to their White counterparts. Over the past 15 years, a number of studies have demonstrated that excessive heat exposure during pregnancy is associated with PTB, with African Americans experiencing disproportionate risk due to structural inequities [ 126 ]. African Americans face greater heat exposure because of lower access to in-home central air conditioning and cooled public spaces compared to their white counterparts. This environmental disparity appears to translate directly into health outcomes. A recent systematic review found that 90% of studies reported a significant association between excessive heat and PTB, with most studies documenting racial disparities in these outcomes [ 127 ]. Variability in heat indices may differentially impact PTB risk between African American and White populations, suggesting that climate factors may be a contributory to health inequities. While differential heat exposure provides a plausible mechanism contributing to persistent PTB disparities between racial groups, researchers note that insufficient studies exist to quantify the precise magnitude of this contribution to overall birth outcome inequities [ 128 ]. This gap in research underscores how environmental factors like heat exposure represent an underexamined pathway through which racial health inequities are maintained and potentially worsened in a warming climate.

Extreme weather events, such as Hurricane Harvey, which struck Houston Texas in August 2017, caused unprecedented flooding in the Houston metropolitan area and resulted in over $125 billion in damages, making it the second costliest disaster in U.S. history. Although prior research has established that disasters adversely impact public health, few population-based studies have examined the specific health effects of Hurricane Harvey. A recent study of residents to assess disparities in physical health, mental health, and health care access in the months following the hurricane revealed that physical health problems and psychological distress, such as post-traumatic stress, disproportionately affected individuals who did not evacuate, African Americans of non-Hispanic origin, older adults, those in households experiencing job loss, and persons with disabilities [ 129 ]. Events like these effectively demonstrate how discrimination and social injustices amplify the health impacts of climate change, which in turn perpetuates inequity.

Digital tools can help bolster resilience to climate impacts and thus potentially ameliorate health inequities that result from climate change. The Lancet Digital Health Commission has explicitly named digital technologies as a fundamental health determinant, on par with income or education, and calls for governance frameworks that ensure equitable benefit sharing and data rights [ 130 ]. Innovations such as low‐bandwidth telehealth for remote communities [ 131 ], a heat early warning system for the elderly [ 132 ], and tools to understand climate change impacts on food security and mental health [ 133 ] are examples of tools that could reduce health inequities. Despite those encouraging developments, the “digital divide” could result in poorer health outcomes in disadvantaged populations despite technological improvements [ 134 ]. Its consequences have already been documented in multiple domains, such as cardiovascular [ 135 ], pulmonary [ 136 ], and kidney disease [ 137 ]. It is therefore important to apply a health equity lens to help inform the equitable design and development of digital health tools [ 138 ].

## 4. Challenges and Limitations

Despite the promise of digital health solutions, several significant barriers hinder their widespread effectiveness. Technological infrastructure disparities—particularly in rural and disadvantaged communities—limit access and equity. Furthermore, concerns about ‘greenwashing’ persist, with some digital health initiatives overstating their environmental benefits without rigorous evidence [ 139 , 140 ]. Compounding these concerns, the ecological costs of digital technology adoption, including increased energy consumption, GHG emissions, resource depletion, habitat destruction, water use, and pollution, are often overlooked [ 141 ].

Rigorous and systematic evaluations are essential to substantiate claims that digital health interventions contribute to environmental sustainability. Currently, the lack of standardized assessment frameworks has impeded the long-term validation and optimization of these technologies. Key barriers—such as inadequate infrastructure, inconsistent outcome measures, and persistent financial constraints—continue to undermine the scalability, reliability, and maturity of digital health innovations. Moreover, the rapid pace of technological development has outstripped the evolution of formal standards and governance frameworks, complicating efforts to measure real-world effectiveness.

Nevertheless, ongoing optimization efforts are strengthening the role of digital health in climate resilience. Enhanced coordination between first responders, healthcare providers, and affected communities is increasingly supported through real-time analytics and telehealth applications. These tools can enable more efficient deployment of personnel and resources during climate-related emergencies, improving response capabilities despite existing limitations.

## 5. Conclusions

Digital health technologies hold considerable promise in addressing climate change. Telemedicine, mobile health applications, AI-driven analytics, and remote monitoring systems offer new pathways for healthcare systems to mitigate GHG emissions, adapt to climate-driven health threats, generate actionable insights, and bridge gaps in health equity. However, realizing this potential requires cautious optimism. Digital health interventions must be critically evaluated to ensure that claims of environmental sustainability are evidence-based rather than aspirational, and persistent disparities in digital access must be addressed to prevent exacerbating existing inequities.

While early evidence indicates that telemedicine and remote monitoring can contribute meaningfully to decarbonization and disaster response, broader systemic challenges remain, including infrastructure vulnerabilities, regulatory barriers, and uneven outcomes across different populations and settings. Additionally, many current applications rely heavily on energy-intensive infrastructure, raising concerns about long-term environmental trade-offs. Given the magnitude of the global climate crisis, it is imperative to accelerate research that bridges digital health and climate change. Future research should prioritize rigorous evaluation of digital health interventions, developing energy-efficient technologies, and integrating climate considerations into digital health policy and practice. By aligning digital innovation with sustainability goals, healthcare systems can move toward a future resilient to climate change and equitable for all populations.
